# Supplementary material for: Evaluation of the Binding Kinetics of RHEB with mTORC1 by In-Cell and In Vitro Assays
Source: Int J Mol Sci. 2021 Aug 16;22(16):8766. doi: 10.3390/ijms22168766 (PMC8395731; doi:10.3390/ijms22168766)
Supplement: Supplementary file 1 [file ijms-22-08766-s001.zip › ijms-1274894-supplementary.pdf]

Supplementary materials for

Evaluation of the Binding Kinetics of RHEB with mTORC1 by In-cell and In vitro Assays

Raef Shams [1,2], Yoshihiro Ito [1,3] and Hideyuki Miyatake [2,3,*]

[1] Emergent Bioengineering Materials Research Team, RIKEN Center for Emergent Matter Science, RIKEN, Wako, Saitama 351-0198, Japan; raef.shams@riken.jp (R.S.); y-ito@riken.jp (Y.I.)
[2] Department of Life Science, Graduate School of Science and Engineering, Saitama University, Saitama city, Saitama 338-8570, Japan; miyatake@riken.jp (H.M.)
[3] Nano Medical Engineering Laboratory, RIKEN Cluster for Pioneering Research, RIKEN, Wako, Saitama 351-0198, Japan
* Correspondence: miyatake@riken.jp; Tel.: + 81-48-467-4979

Content:

1- Supplementary notes:

1.1. Human mTOR gene sequence (7647 bp)
1.2. Human RHEB gene sequence (507 bp)

2- Supplementary figures:

2.1. Figure S1. On-lysosome activation of mTORC1 by RHEB.
2.2. Figure S2. Construction and purification of overexpressed mTOR.
2.3. Figure S3. Construction and purification of overexpressed RHEB.
2.4. Figure S4. The established AlphaLISA method for RHEB–mTOR interaction.
2.5. Figure S5. The constructed plasmids for NanoBiT assay.
2.6. Figure S6. Construction and purification of overexpressed ΔN-FAT-M.
2.7. Figure S7. Construction and purification of overexpressed ΔN.
2.8. Figure S8. Construction and purification of overexpressed ΔATP.
2.9. Figure S9. Schematic illustration showing BLItz measurement steps.

Note 1. Human mTOR gene sequence (7647 bp)
(http://www.kazusa.or.jp/kop/vd/FHC01207/)

(https://www.uniprot.org/uniprot/P42345)

Red highlights indicate gene sequences of the selected fragments of RHEB binding site

```
   1 ATGCTTGGAACCGGACCTGCCGCCGCCACCACCGCTGCCACCACATCTAGCAATGTGAGC
  61 GTCCTGCAGCAGTTTGCCAGTGGCCTAAAGAGCCGGAATGAGGAAACCAGGGCCAAAGCC
 121 GCCAAGGAGCTCCAGCACTATGTCACCATGGAACTCCGAGAGATGAGTCAAGAGGAGTCT N-heat
                                                               S    60
 181 ACTCGCTTCTATGACCAACTGAACCATCACATTTTTGAATTGGTTTCCAGCTCAGATGCC
      T  R  F  Y  D  Q  L  N  H  H  I  F  E  L  V  S  S  S  D  A    80
 241 AATGAGAGGAAAGGTGGCATCTTGGCCATAGCTAGCCTCATAGGAGTGGAAGGTGGGAAT
      N  E  R  K  G  G  I  L  A  I  A  S  L  I  G  V  E  G  G  N   100
 301 GCCACCCGAATTGGCAGATTTGCCAACTATCTTCGGAACCTCCTCCCCTCCAATGACCCA
      A  T  R  I  G  R  F  A  N  Y  L  R  N  L  L  P  S  N  D  P   120
 361 GTTGTCATGGAAATGGCATCCAAGGCCATTGGCCGTCTTGCCATGGCAGGGGACACTTTT
      V  V  M  E  M  A  S  K  A  I  G  R  L  A  M  A  G  D  T  F   140
 421 ACCGCTGAGTACGTGGAATTTGAGGTGAAGCGAGCCCTGGAATGGCTGGGTGCTGACCGC
      T  A  E  Y  V  E  F  E  V  K  R  A  L  E  W  L  G  A  D  R   160
 481 AATGAGGGCCGGAGACATGCAGCTGTCCTGGTTCTCCGTGAGCTGGCCATCAGCGTCCCT
      N  E  G  R  R  H  A                                          180
 541 ACCTTCTTCTTCCAGCAAGTGCAACCCTTCTTTGACAACATTTTTGTGGCCGTGTGGGAC
 601 CCCAAACAGGCCATCCGTGAGGGAGCTGTAGCCGCCCTTCGTGCCTGTCTGATTCTCACA
 661 ACCCAGCGTGAGCCGAAGGAGATGCAGAAGCCTCAGTGGTACAGGCACACATTTGAAGAA
 721 GCAGAGAAGGGATTTGATGAGACCTTGGCCAAAGAGAAGGGCATGAATCGGGATGATCGG
 781 ATCCATGGAGCCTTGTTGATCCTTAACGAGCTGGTCCGAATCAGCAGCATGGAGGGAGAG
 841 CGTCTGAGAGAAGAAATGGAAGAAATCACACAGCAGCAGCTGGTACACGACAAGTACTGC
 901 AAAGATCTCATGGGCTTCGGAACAAAACCTCGTCACATTACCCCCTTCACCAGTTTCCAG
 961 GCTGTACAGCCCCAGCAGTCAAATGCCTTGGTGGGGCTGCTGGGGTACAGCTCTCACCAA
1021 GGCCTCATGGGATTTGGGACCTCCCCCAGTCCAGCTAAGTCCACCCTGGTGGAGAGCCGG
1081 TGTTGCAGAGACTTGATGGAGGAGAAATTTGATCAGGTGTGCCAGTGGGTGCTGAAATGC
1141 AGGAATAGCAAGAACTCGCTGATCCAAATGACAATCCTTAATTTGTTGCCCCGCTTGGCT
1201 GCATTCCGACCTTCTGCCTTCACAGATACCCAGTATCTCCAAGATACCATGAACCATGTC
1261 CTAAGCTGTGTCAAGAAGGAGAAGGAACGTACAGCGGCCTTCCAAGCCCTGGGGCTACTT
1321 TCTGTGGCTGTGAGGTCTGAGTTTAAGGTCTATTTGCCTCGCGTGCTGGACATCATCCGA
1381 GCGGCCCTGCCCCCAAAGGACTTCGCCCATAAGAGGCAGAAGGCAATGCAGGTGGACGCC
1441 ACAGTCTTCACTTGCATCAGCATGCTGGCTCGAGCAATGGGGCCAGGCATCCAGCAGGAT
1501 ATCAAGGAGCTGCTGGAGCCCATGCTGGCAGTGGGACTAAGCCCTGCCCTCACTGCAGTG
1561 CTCTACGACCTGAGCCGTCAGATTCCACAGCTAAAGAAGGACATTCAAGATGGGCTACTG
1621 AAAATGCTGTCCCTGGTCCTTATGCACAAACCCCTTCGCCACCCAGGCATGCCCAAGGGC
1681 CTGGCCCATCAGCTGGCCTCTCCTGGCCTCACGACCCTCCCTGAGGCCAGCGATGTGGGC
1741 AGCATCACTCTTGCCCTCCGAACGCTTGGCAGCTTTGAATTTGAAGGCCACTCTCTGACC
1801 CAATTTGTTCGCCACTGTGCGGATCATTTCCTGAACAGTGAGCACAAGGAGATCCGCATG
1861 GAGGCTGCCCGCACCTGCTCCCGCCTGCTCACACCCTCCATCCACCTCATCAGTGGCCAT
1921 GCTCATGTGGTTAGCCAGACCGCAGTGCAAGTGGTGGCAGATGTGCTTAGCAAACTGCTC
1981 GTAGTTGGGATAACAGATCCTGACCCTGACATTCGCTACTGTGTCTTGGCGTCCCTGGAC
2041 GAGCGCTTTGATGCACACCTGGCCCAGGCGGAGAACTTGCAGGCCTTGTTTGTGGCTCTG
2101 AATGACCAGGTGTTTGAGATCCGGGAGCTGGCCATCTGCACTGTGGGCCGACTCAGTAGC
2161 ATGAACCCTGCCTTTGTCATGCCTTTCCTGCGCAAGATGCTCATCCAGATTTTGACAGAG
2221 TTGGAGCACAGTGGGATTGGAAGAATCAAAGAGCAGAGTGCCCGCATGCTGGGGCACCTG
2281 GTCTCCAATGCCCCCCGACTCATCCGCCCCTACATGGAGCCTATTCTGAAGGCATTAATT
2341 TTGAAACTGAAAGATCCAGACCCTGATCCAAACCCAGGTGTGATCAATAATGTCCTGGCA
2401 ACAATAGGAGAATTGGCACAGGTTAGTGGCCTGGAAATGAGGAAATGGGTTGATGAACTT
2461 TTTATTATCATCATGGACATGCTCCAGGATTCCTCTTTGTTGGCCAAAAGGCAGGTGGCT
2521 CTGTGGACCCTGGGACAGTTGGTGGCCAGCACTGGCTATGTAGTAGAGCTCTACAGGAAG
2581 TACCCTACTTTGCTTGAGGTGCTACTGAATTTTCTGAAGACTGAGCAGAACCAGGGTACA
2641 CGCAGAGAGGCCATCCGTGTGTTAGGGCTTTTAGGGGCTTTGGATCCTTACAAGCACAAA
2701 GTGAACATTGGCATGATAGACCAGTCCCGGGATGCCTCTGCTGTCAGCCTGTCAGAATCC
```

```
2761 AAGTCAAGTCAGGATTCCTCTGACTATAGCACTAGTGAAATGCTGGTCAACATGGGAAAC
2821 TTGCCTCTGGATGAGTTCTACCCAGCTGTGTCCATGGTGGCCCTGATGCGGATCTTCCGA
2881 GACCAGTCACTCTCTCATCATCACACCATGGTTGTCCAGGCCATCACCTTCATCTTCAAG M-heat
                       H  H  T  M  V  V  Q  A  I  T  F  I  F  K    980
2941 TCCCTGGGACTCAAATGTGTGCAGTTCCTGCCCCAGGTCATGCCCACGTTCCTTAATGTC
      S  L  G  L  K  C  V  Q  F  L  P  Q  V  M  P  T  F  L  N  V   1000
3001 ATTCGAGTCTGTGATGGGGCCATCCGGGAATTTTTGTTCCAGCAGCTGGGAATGTTGGTG
      I  R  V  C  D  G  A  I  R  E  F  L  F  Q  Q  L  G  M  L  V   1020
3061 TCCTTTGTGAAGAGCCACATCAGACCTTATATGGATGAAATAGTCACCCTCATGAGAGAA
      S  F  M                                                      1040
3121 TTCTGGGTCATGAACACCTCAATTCAGAGCACGATCATTCTTCTCATTGAGCAAATTGTG
3181 GTAGCTCTTGGGGGTGAATTTAAGCTCTACCTGCCCCAGCTGATCCCACACATGCTGCGT
3241 GTCTTCATGCATGACAACAGCCCAGGCCGCATTGTCTCTATCAAGTTACTGGCTGCAATC
3301 CAGCTGTTTGGCGCCAACCTGGATGACTACCTGCATTTACTGCTGCCTCCTATTGTTAAG
3361 TTGTTTGATGCCCCTGAAGCTCCACTGCCATCTCGAAAGGCAGCGCTAGAGACTGTGGAC
3421 CGCCTGACGGAGTCCCTGGATTTCACTGACTATGCCTCCCGGATCATTCACCCTATTGTT
3481 CGAACACTGGACCAGAGCCCAGAACTGCGCTCCACAGCCATGGACACGCTGTCTTCACTT
3541 GTTTTTCAGCTGGGGAAGAAGTACCAAATTTTCATTCCAATGGTGAATAAAGTTCTGGTG
3601 CGACACCGAATCAATCATCAGCGCTATGATGTGCTCATCTGCAGAATTGTCAAGGGATAC
3661 ACACTTGCTGATGAAGAGGAGGATCCTTTGATTTACCAGCATCGGATGCTTAGGAGTGGC  FAT
                                                              G   1240
3721 CAAGGGGATGCATTGGCTAGTGGACCAGTGGAAACAGGACCCATGAAGAAACTGCACGTC
      Q  G  D  A  L  A  S  G  P  V  E  T  G  P  M  K  K  L  H  V   1260
3781 AGCACCATCAACCTCCAAAAGGCCTGGGGCGCTGCCAGGAGGGTCTCCAAAGATGACTGG
      S  T  I  N  L  Q  K  A  W  G  A  A  R  R  V  S  K  D  D  W   1280
3841 CTGGAATGGCTGAGACGGCTGAGCCTGGAGCTGCTGAAGGACTCATCATCGCCCTCCCTG
      L  E  W  L  R  R  L  S  L  E  L  L  K  D  S  S  S  P  S  L   1300
3901 CGCTCCTGCTGGGCCCTGGCACAGGCCTACAACCCGATGGCCAGGGATCTCTTCAATGCT
      R  S  C  W  A  L  A  Q  A  Y  N  P  M  A  R  D  L  F  N  A   1320
3961 GCATTTGTGTCCTGCTGGTCTGAACTGAATGAAGATCAACAGGATGAGCTCATCAGAAGC
      A  F  V  S  C  W  S  E  L  N  E  D  Q  Q  D  E  L  I  R  S   1340
4021 ATCGAGTTGGCCCTCACCTCACAAGACATCGCTGAAGTCACACAGACCCTCTTAAACTTG
      I  E  L  A  L  T  S  Q  D  I  A  E  V  T  Q  T  L  L  N  L   1360
4081 GCTGAATTCATGGAACACAGTGACAAGGGCCCCCTGCCACTGAGAGATGACAATGGCATT
4141 GTTCTGCTGGGTGAGAGAGCTGCCAAGTGCCGAGCATATGCCAAAGCACTACACTACAAA
4201 GAACTGGAGTTCCAGAAAGGCCCCACCCCTGCCATTCTAGAATCTCTCATCAGCATTAAT
4261 AATAAGCTACAGCAGCCGGAGGCAGCGGCCGGAGTGTTAGAATATGCCATGAAACACTTT
4321 GGAGAGCTGGAGATCCAGGCTACCTGGTATGAGAAACTGCACGAGTGGGAGGATGCCCTT
4381 GTGGCCTATGACAAGAAAATGGACACCAACAAGGACGACCCAGAGCTGATGCTGGGCCGC
4441 ATGCGCTGCCTCGAGGCCTTGGGGGAATGGGGTCAACTCCACCAGCAGTGCTGTGAAAAG
4501 TGGACCCTGGTTAATGATGAGACCCAAGCCAAGATGGCCCGGATGGCTGCTGCAGCTGCA
4561 TGGGGTTTAGGTCAGTGGGACAGCATGGAAGAATACACCTGTATGATCCCTCGGGACACC
4621 CATGATGGGGCATTTTATAGAGCTGTGCTGGCACTGCATCAGGACCTCTTCTCCTTGGCA
4681 CAACAGTGCATTGACAAGGCCAGGGACCTGCTGGATGCTGAATTAACTGCAATGGCAGGA
4741 GAGAGTTACAGTCGGGCATATGGGGCCATGGTTTCTTGCCACATGCTGTCCGAGCTGGAG
4801 GAGGTTATCCAGTACAAACTTGTCCCCGAGCGACGAGAGATCATCCGCCAGATCTGGTGG
4861 GAGAGACTGCAGGGCTGCCAGCGTATCGTAGAGGACTGGCAGAAAATCCTTATGGTGCGG
4921 TCCCTTGTGGTCAGCCCTCATGAAGACATGAGAACCTGGCTCAAGTATGCAAGCCTGTGC
4981 GGCAAGAGTGGCAGGCTGGCTCTTGCTCATAAAACTTTAGTGTTGCTCCTGGGAGTTGAT
5041 CCGTCTCGGCAACTTGACCATCCTCTGCCAACAGTTCACCCTCAGGTGACCTATGCCTAC
5101 ATGAAAAACATGTGGAAGAGTGCCCGCAAGATCGATGCCTTCCAGCACATGCAGCATTTT
5161 GTCCAGACCATGCAGCAACAGGCCCAGCATGCCATCGCTACTGAGGACCAGCAGCATAAG
5221 CAGGAACTGCACAAGCTCATGGCCCGATGCTTCCTGAAACTTGGAGAGTGGCAGCTGAAT
5281 CTACAGGGCATCAATGAGAGCACAATCCCCAAAGTGCTGCAGTACTACAGCGCCGCCACA
5341 GAGCACGACCGCAGCTGGTACAAGGCCTGGCATGCGTGGGCAGTGATGAACTTCGAAGCT
5401 GTGCTACACTACAAACATCAGAACCAAGCCCGCGATGAGAAGAAGAAACTGCGTCATGCC
5461 AGCGGGGCCAACATCACCAACGCCACCACTGCCGCCACCACGGCCGCCACTGCCACCACC
5521 ACTGCCAGCACCGAGGGCAGCAACAGTGAGAGCGAGGCCGAGAGCACCGAGAACAGCCCC
5581 ACCCCATCGCCGCTGCAGAAGAAGGTCACTGAGGATCTGTCCAAAACCCTCCTGATGTAC
5641 ACGGTGCCTGCCGTCCAGGGCTTCTTCCGTTCCATCTCCTTGTCACGAGGCAACAACCTC
5701 CAGGATACACTCAGAGTTCTCACCTTATGGTTTGATTATGGTCACTGGCCAGATGTCAAT
```

```
5761 GAGGCCTTAGTGGAGGGGGTGAAAGCCATCCAGATTGATACCTGGCTACAGGTTATACCT
5821 CAGCTCATTGCAAGAATTGATACGCCCAGACCCTTGGTGGGACGTCTCATTCACCAGCTT
5881 CTCACAGACATTGGTCGGTACCACCCCCAGGCCCTCATCTACCCACTGACAGTGGCTTCT
5941 AAGTCTACCACGACAGCCCGGCACAATGCAGCCAACAAGATTCTGAAGAACATGTGTGAG
6001 CACAGCAACACCCTGGTCCAGCAGGCCATGATGGTGAGCGAGGAGCTGATCCGAGTGGCC
6061 ATCCTCTGGCATGAGATGTGGCATGAAGGCCTGGAAGAGGCATCTCGTTTGTACTTTGGG
6121 GAAAGGAACGTGAAAGGCATGTTTGAGGTGCTGGAGCCCTTGCATGCTATGATGGAACGG
6181 GGCCCCCAGACTCTGAAGGAAACATCCTTTAATCAGGCCTATGGTCGAGATTTAATGGAG
6241 GCCCAAGAGTGGTGCAGGAAGTACATGAAATCAGGGAATGTCAAGGACCTCACCCAAGCC
6301 TGGGACCTCTATTATCATGTGTTCCGACGAATCTCAAAGCAGCTGCCTCAGCTCACATCC
6361 TTAGAGCTGCAATATGTTTCCCCAAAACTTCTGATGTGCCGGGACCTTGAATTGGCTGTG
6421 CCAGGAACATATGACCCCAACCAGCCAATCATTCGCATTCAGTCCATAGCACCGTCTTTG ATP site
                            Q  P  I  I  R  I  Q  S  I  A  P  S  L   2160
6481 CAAGTCATCACATCCAAGCAGAGGCCCCGGAAATTGACACTTATGGGCAGCAACGGACAT
      Q  V  I  T  S  K  Q  R  P  R  K  L  T  L  M  G  S  N  G  H   2180
6541 GAGTTTGTTTTCCTTCTAAAAGGCCATGAAGATCTGCGCCAGGATGAGCGTGTGATGCAG
      E  F  V  F  L  L  K  G  H  E  D  L  R  Q  D  E  R  V  M  Q   2200
6601 CTCTTCGGCCTGGTTAACACCCTTCTGGCCAATGACCCAACATCTCTTCGGAAAAACCTC
      L  F  G  L  V  N  T  L  L  A  N  D  P  T  S  L  R  K  N  L   2220
6661 AGCATCCAGAGATACGCTGTCATCCCTTTATCGACCAACTCGGGCCTCATTGGCTGGGTT
      S  I  Q  R  Y  A  V  I  P  L  S  T  N  S  G  L  I  G  W  V   2240
6721 CCCCACTGTGACACACTGCACGCCCTCATCCGGGACTACAGGGAGAAGAAGAAGATCCTT
      P  H  C  D  T  L  H  A  L  I  R  D  Y  R  E  K  K  K  I  L   2260
6781 CTCAACATCGAGCATCGCATCATGTTGCGGATGGCTCCGGACTATGACCACTTGACTCTG
      L  N  I  E  H  R  I  M  L  R  M  A  P  D  Y  D  H  L  T  L   2280
6841 ATGCAGAAGGTGGAGGTGTTTGAGCATGCCGTCAATAATACAGCTGGGGACGACCTGGCC
      M  Q  K  V  E  V  F  E  H  A  V  N  N  T  A  G  D  D  L  A   2300
6901 AAGCTGCTGTGGCTGAAAAGCCCCAGCTCCGAGGTGTGGTTTGACCGAAGAACCAATTAT
6961 ACCCGTTCTTTAGCGGTCATGTCAATGGTTGGGTATATTTTAGGCCTGGGAGATAGACAC
7021 CCATCCAACCTGATGCTGGACCGTCTGAGTGGGAAGATCCTGCACATTGACTTTGGGGAC
7081 TGCTTTGAGGTTGCTATGACCCGAGAGAAGTTTCCAGAGAAGATTCCATTTAGACTAACA
7141 AGAATGTTGACCAATGCTATGGAGGTTACAGGCCTGGATGGCAACTACAGAATCACATGC
7201 CACACAGTGATGGAGGTGCTGCGAGAGCACAAGGACAGTGTCATGGCCGTGCTGGAAGCC
7261 TTTGTCTATGACCCCTTGCTGAACTGGAGGCTGATGGACACAAATACCAAAGGCAACAAG
7321 CGATCCCGAACGAGGACGGATTCCTACTCTGCTGGCCAGTCAGTCGAAATTTTGGACGGT
7381 GTGGAACTTGGAGAGCCAGCCCATAAGAAAACGGGGACCACAGTGCCAGAATCTATTCAT
7441 TCTTTCATTGGAGACGGTTTGGTGAAACCAGAGGCCCTAAATAAGAAAGCTATCCAGATT
7501 ATTAACAGGGTTCGAGATAAGCTCACTGGTCGGGACTTCTCTCATGATGACACTTTGGAT
7561 GTTCCAACGCAAGTTGAGCTGCTCATCAAACAAGCGACATCCCATGAAAACCTCTGCCAG
7621 TGCTATATTGGCTGGTGCCCTTTCTGG
```

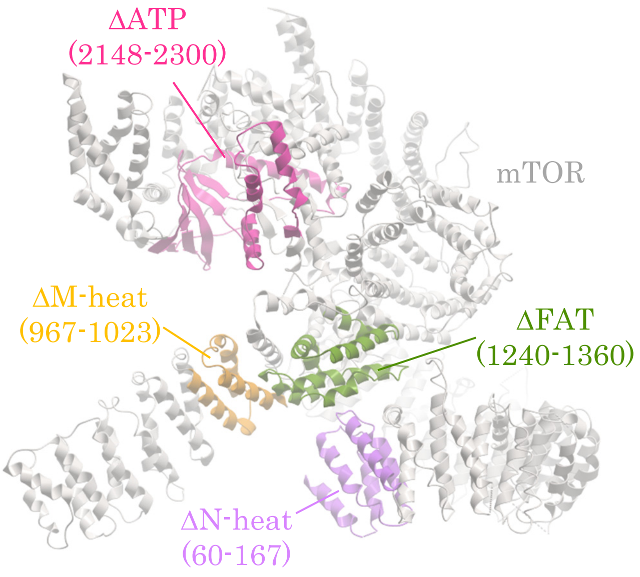

Note 2. Human RHEB gene sequence (507 bp)

(https://www.uniprot.org/uniprot/Q15382)

```
  1 CAGTCCAAAAGCCGCAAAATCGCCATTCTTGGCTATCGCTCTGTTGGCAAATCGAGTCTG
 61 ACCATTCAGTTCGTTGAAGGCCAATTCGTTGATTCCTATGATCCGACCATTGAAAACACC
121 TTCACGAAACTCATTACGGTTAATGGACAGGAATACCATCTGCAGTTAGTGGATACAGCT
181 GGTCAAGACGAGTACAGCATCTTTCCACAGACCTATTCGATTGACATCAATGGCTACATT
241 CTGGTGTATAGTGTCACTTCGATCAAAAGCTTTGAAGTGATCAAGGTGATTCATGGGAAA
301 CTGCTGGATATGGTGGGTAAAGTGCAGATTCCGATCATGCTGGTAGGTAACAAGAAAGAC
361 TTGCACATGGAACGTGTCATCTCATATGAAGAGGGGAAAGCTTTAGCCGAATCTTGGAAT
421 GCGGCATTTCTGGAGAGCTCAGCGAAAGAGAACCAAACTGCAGTAGATGTCTTTCGTCGC
481 ATTATTCTGGAAGCGGAAAAGTTGGAA
```

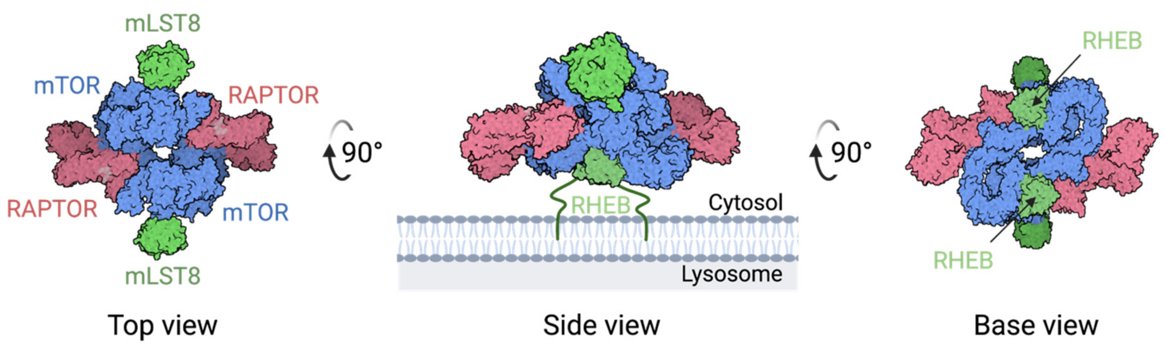

Figure S1: On-lysosome activation of mTORC1 by RHEB. Illustration created by BioRender (https://app.biorender.com/).

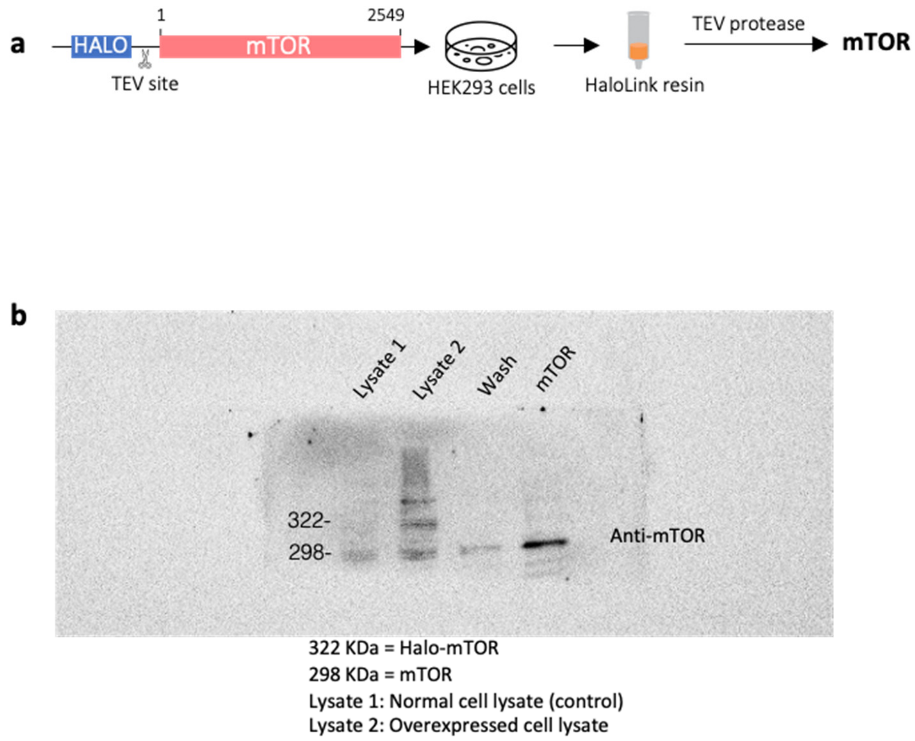

Figure S2. Construction and purification of overexpressed mTOR. (**a**) Schematic illustration showing the Halo-tagged mTOR construct in pFN21A vector which was transfected into HEK293 cells and purified by HaloLink resin. TEV site (EDLYFQ ↓ S) is a cleavage site for TEV protease. (**b**) Whole view of Western blot analysis of the different stages of mTOR purification showing the expression of the Halo-tagged mTOR.

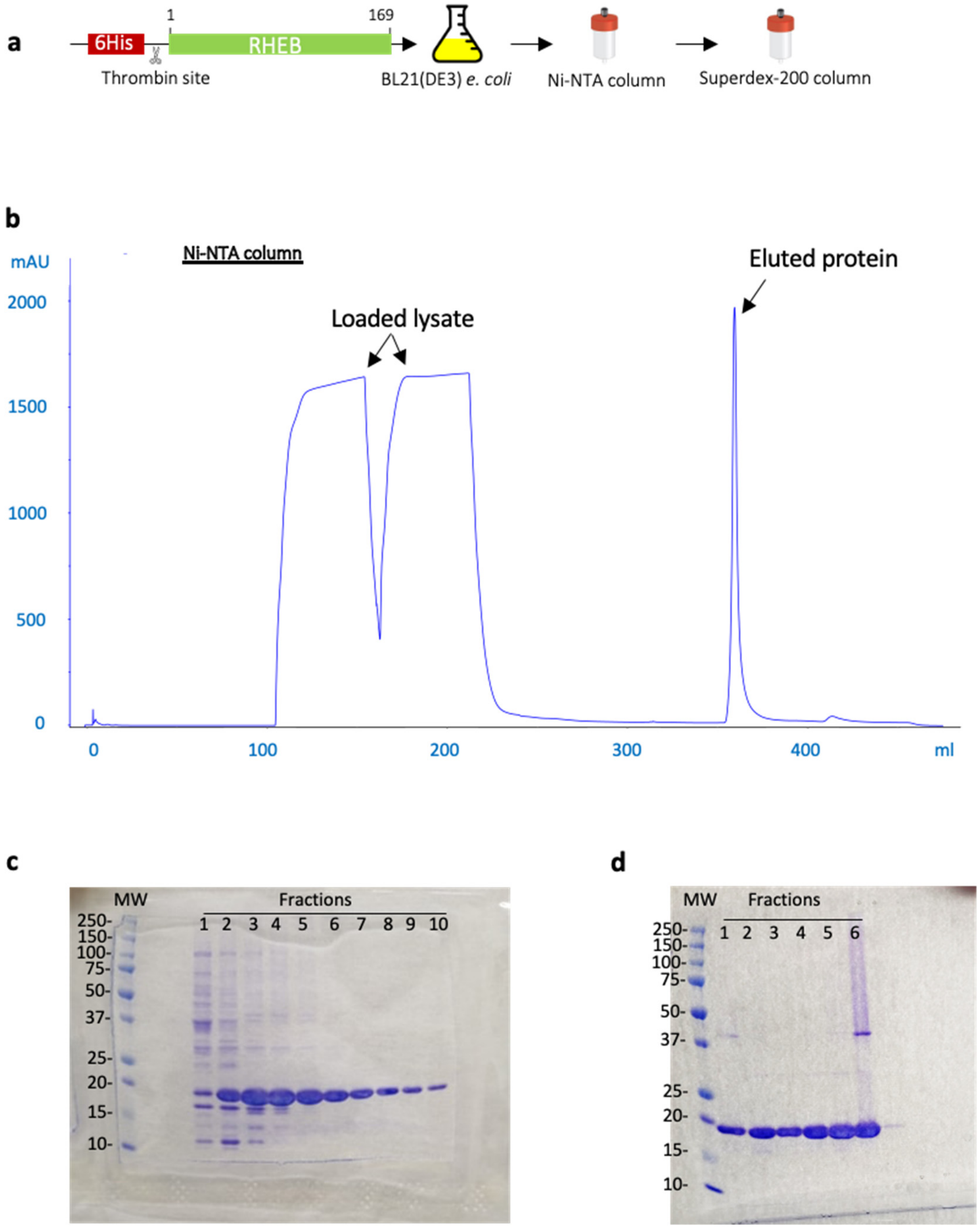

Figure S3. Construction and purification of overexpressed RHEB (aa 1-169). (**a**) Schematic illustration showing the 6His-tagged RHEB construct in pET15b vector which was transformed into BL21(DE3) E. coli and purified by Ni-NTA and Superdex-200 columns. (**b**) FPLC chromatogram of RHEB purification by Ni-NTA column. (**c**) Coomassie brilliant blue R-250 stained SDS-PAGE of the eluted fractions of RHEB (~20 kDa) from Ni-NTA column. The contaminating bands of fractions 1-4 were due to the molecules nonspecifically bound on the Ni-NTA column. Fractions 5-10 were used for Superdex-200 purification. (**d**) Full image SDS-PAGE (stained with Coomassie brilliant blue R-250) of the eluted fractions of RHEB protein from Superdex-200 column. Fractions 1 and 6 contain mixture of RHEB monomer and dimer. Fractions 2-5 were used as a final RHEB product.

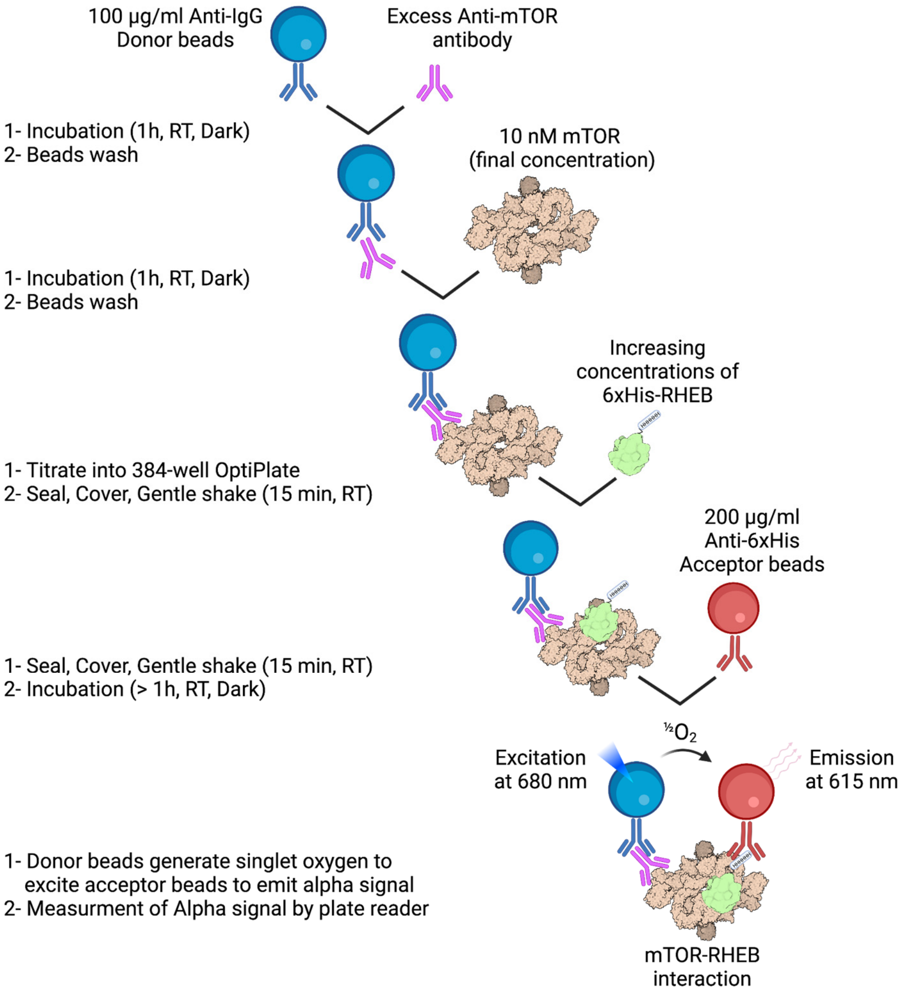

Figure S4. The established AlphaLISA method used to determine the binding kinetics of RHEB-mTOR interaction. RT, room temperature. Illustration created by BioRender (https://app.biorender.com/).

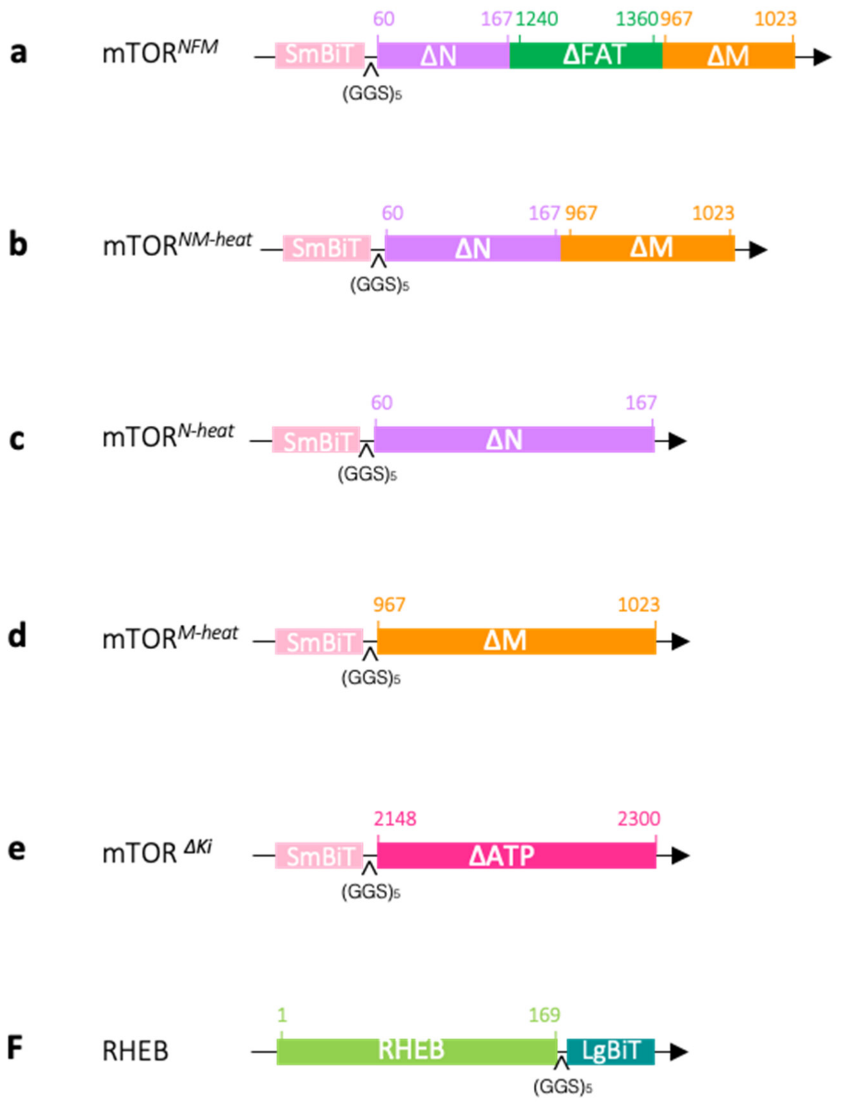

Figure S5. Plasmids of the indicated constructs for NanoBiT assay showing the SmBiT subunit at the N-terminus and the LgBiT subunit of RHEB at the C-terminus. $(GGS)_5$ is five repeats of GGS amino acid sequence as a linker between the protein of interest and the SmBiT or LgBiT.

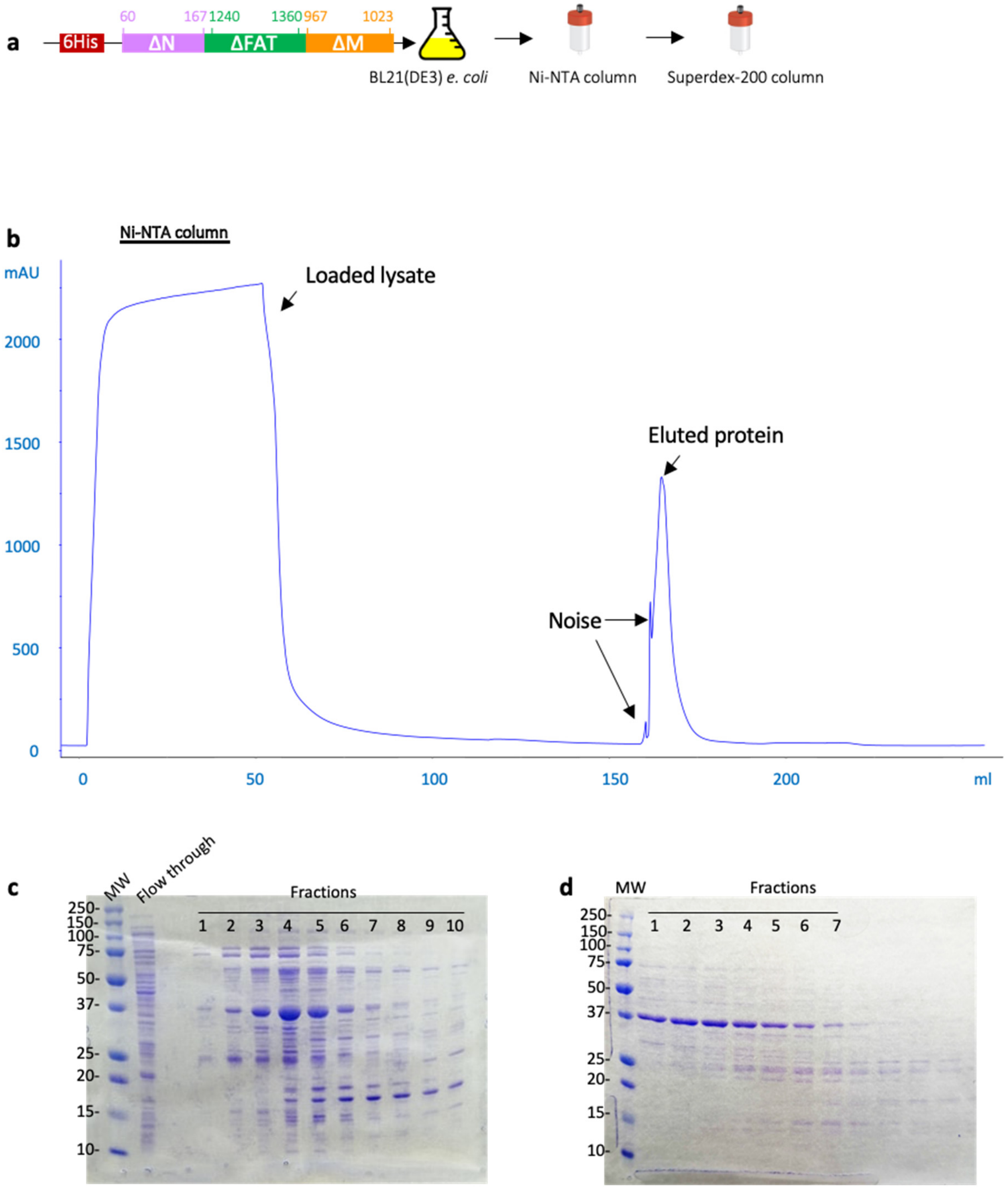

Figure S6. Construction and purification of overexpressed ΔN-FAT-M (aa 60-167, aa 1240-1360, aa 967-1023). (**a**) Schematic illustration showing the 6His-tagged ΔN-FAT-M construct in pET15b vector which was transformed into BL21(DE3) E. coli and purified by Ni-NTA and Superdex-200 columns. (**b**) FPLC chromatogram of ΔN-FAT-M purification by Ni-NTA column. Loaded lysate is a flow through without binding. (**c**) Coomassie brilliant blue R-250 stained SDS-PAGE of the eluted fractions of ΔN-FAT-M (~37.5 kDa) from Ni-NTA column. Fractions 1-6 were used for Superdex-200 purification. (**d**) Full image SDS-PAGE (stained with Coomassie brilliant blue R-250) of the eluted fractions of ΔN-FAT-M protein from Superdex-200 column. Fractions 1-4 were used as a final ΔN-FAT-M product. The contaminating bands were probably derived from protein oligomerization.

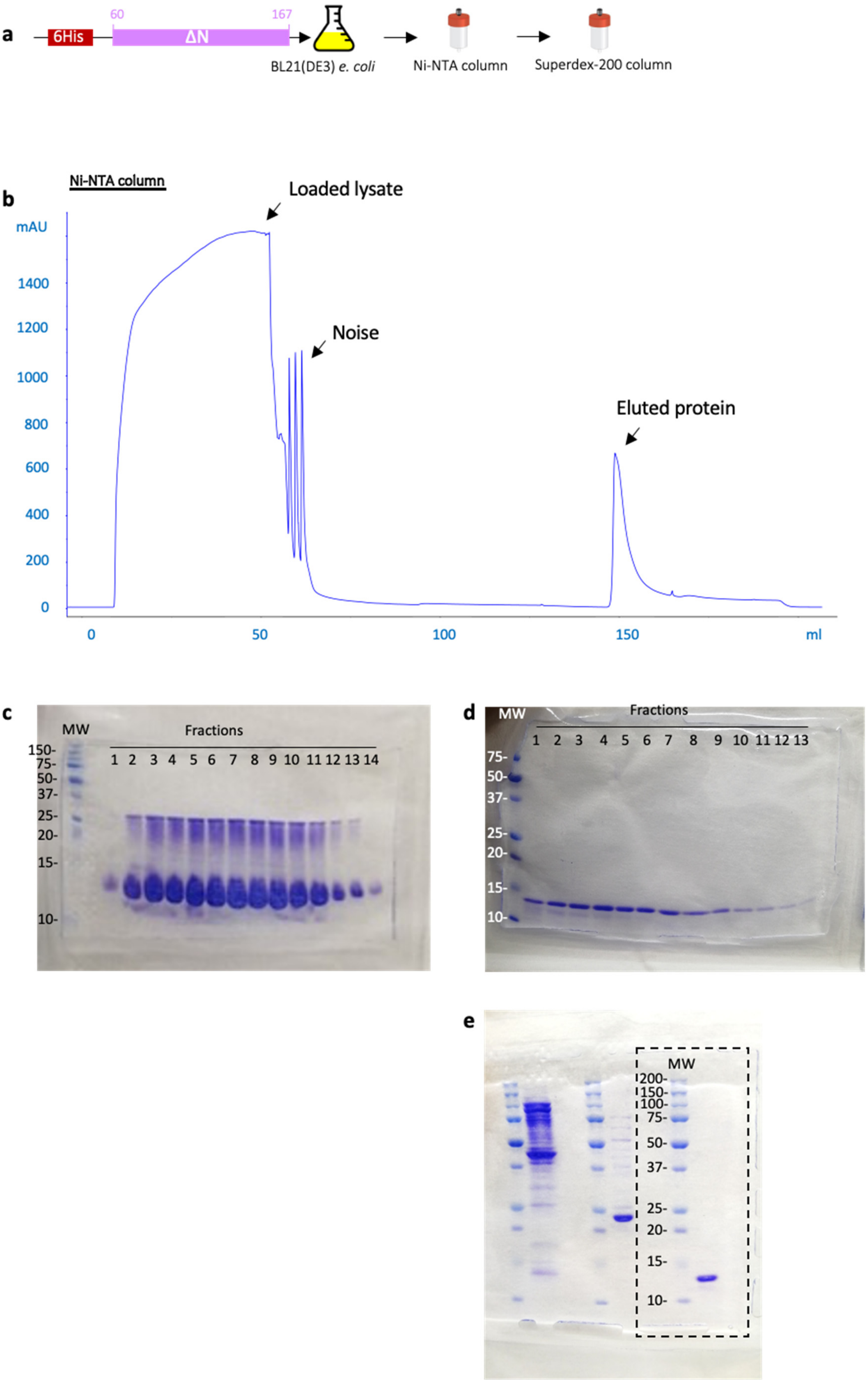
a
6His
60
ΔN
167
BL21(DE3) e. coli
Ni-NTA column
Superdex-200 column
b
Ni-NTA column
mAU
Loaded lysate
Noise
Eluted protein
1400
1200
1000
800
600
400
200
0
0
50
100
150
ml
c
MW
Fractions
1 2 3 4 5 6 7 8 9 10 11 12 13 14
150-
75-
50-
37-
25-
20-
15-
10-
d
MW
Fractions
1 2 3 4 5 6 7 8 9 10 11 12 13
75-
50-
37-
25-
20-
15-
10-
e
MW
200-
150-
100-
75-
50-
37-
25-
20-
15-
10-

Figure S7. Construction and purification of overexpressed ΔN (60-167). (**a**) Schematic illustration showing the 6His-tagged ΔN construct in pET15b vector which was transformed into BL21(DE3) E. coli and purified by Ni-NTA and Superdex-200 columns. (**b**) FPLC chromatogram of ΔN purification by Ni-NTA column. Loaded lysate is a flow through without binding. (**c**) Coomassie brilliant blue R-250 stained SDS-PAGE of the eluted fractions of ΔN (~13.3 kDa) from Ni-NTA column. Fractions 1-14 containing mixture of monomer and dimer were used for Superdex-200 purification. Loaded lysate is a flow through without binding. (**d**) Full image of SDS-PAGE (stained with Coomassie brilliant blue R-250) of the eluted fractions of ΔN protein eluted from Superdex-200 column. (**e**) Full image of SDS-PAGE showing the final product of ΔN highlighted by the dotted square. The other bands are for another studies.

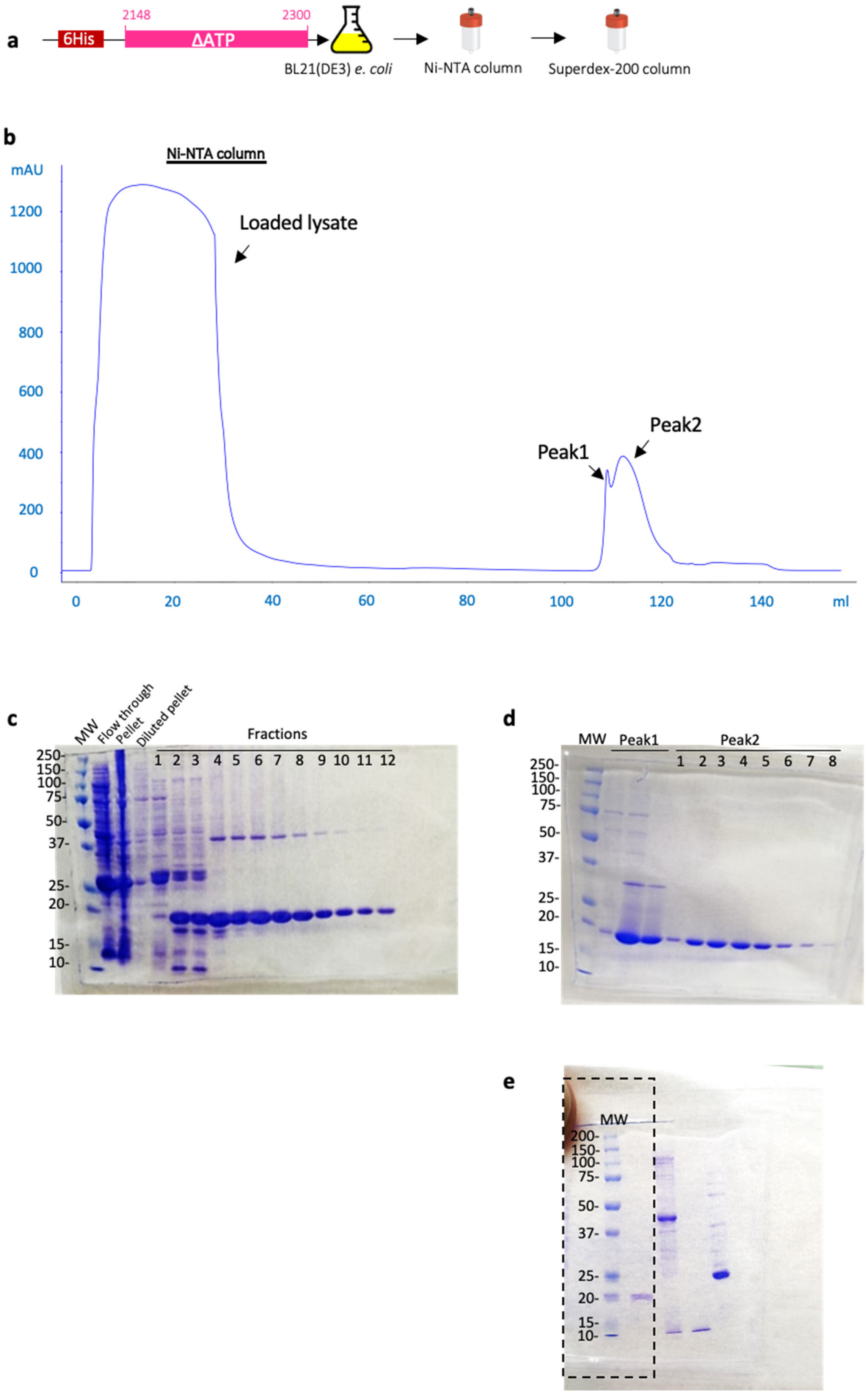
a
6His
2148
ΔATP
2300
BL21(DE3) e. coli
Ni-NTA column
Superdex-200 column
b
Ni-NTA column
mAU
Loaded lysate
Peak1
Peak2
ml
c
MW
Flow through
Pellet
Diluted pellet
Fractions
d
MW
Peak1
Peak2
e
MW

Figure S8. Construction and purification of overexpressed ΔATP (2148-2300). (**a**) Schematic illustration showing the 6His-tagged ΔATP construct in pET15b vector which was transformed into BL21(DE3) E. coli and purified by Ni-NTA and Superdex-200 columns. (**b**) FPLC chromatogram of ΔATP purification by Ni-NTA column. Loaded lysate is a flow through without binding. (**c**) Full image of SDS-PGE (stained with Coomassie brilliant blue R-250) of the fractions of ΔATP (~20 kDa) eluted from Ni-NTA column. The contaminating bands of fractions 1-3 were due to the nonspecific binding on Ni-NTA column. Fractions 4-12 contain mixture of monomer and dimer were used for Superdex-200 purification. (**d**) Full image of SDS-PAGE (stained with Coomassie brilliant blue R-250) of the eluted fractions of ΔATP protein from Superdex-200 column. Peak1 fractions were for protein oligomers while peak2 fraction were for ΔATP monomer. (**e**) Full image of SDS-PAGE showing the final product of ΔATP highlighted by the dotted square. The other bands are for another studies.

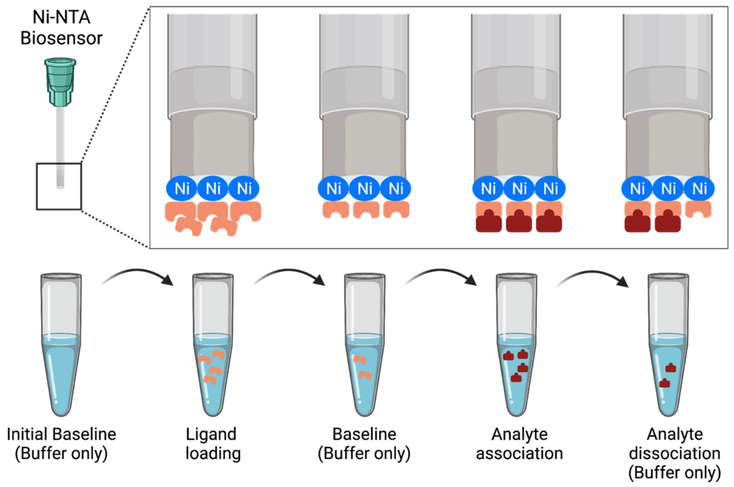

Figure S9. Schematic illustration showing the BLItz measurement steps of protein-protein interactions. The experiments were performed with shaking at 2000 rpm at 25 °C. The illustration was created by BioRender (https://app.biorender.com/).
